# Perspectives and implications of the Improving Coverage Measurement Core Group’s validation studies for household surveys

**DOI:** 10.7189/jogh.08.010606

**Published:** 2018-06

**Authors:** Fred Arnold, Shane M Khan

**Affiliations:** 1 Demographic and Health Surveys Program, ICF, Rockville, Maryland, USA; 2 Data & Analytics, Division of Data, Research and Policy, UNICEF, New York, New York, USA

## Abstract

**Background:**

Formal validation studies are of critical importance in determining whether or not household survey questions are providing accurate information on what they intend to measure. These studies supplement an array of methods used to evaluate survey questions.

**Methods and Findings:**

This paper summarizes the methods used by the two major international household survey programmes – The Demographic and Health Surveys Program (DHS) and the Multiple Indicator Cluster Surveys (MICS) – to decide on possible modifications to the survey questions, nomenclature, tables, and interpretation of findings over time as additional information on the validity of the questions becomes available.

**Conclusions:**

Validation studies are most useful if they are conducted in a variety of different settings in low- and middle-income countries, preferably using representative samples and procedures that replicate DHS and MICS field conditions. Pilot tests, pre-tests in each country, feedback from interviewers and survey staff, and cognitive interviewing provide additional information about how well survey questions are understood and provide accurate information. The paper provides specific examples of changes that have been made in response to findings from validation studies and changes in international recommendations.

Quantitative data are key to monitoring national and global-level commitments, as well as for prioritizing, planning, and evaluating programmes at the country level. For the Millennium Development Goals (MDGs), comparable data on a range of indicators were made available, in large part through nationally-representative household surveys. The UNICEF-supported Multiple Indicator Cluster Surveys (MICS) and the USAID-supported Demographic and Health Surveys (DHS) provided a substantial proportion of the data required to monitor progress toward the MDGs. For example, at the endline assessment for the MDGs, DHS and MICS contributed about half of the data on contraceptive prevalence, 60 percent of the data on child malnutrition, and 80 percent of the data on children’s use of insecticide-treated mosquito nets [1]. It is widely expected that household surveys will continue to be indispensable for tracking Sustainable Development Goals (SDGs) indicators. Further, numerous global frameworks, such as the Every Newborn Action Plan (ENAP) and the Countdown to 2030 have also developed or use sector-specific indicators, many of which rely heavily on household surveys as their source of data on the general household population.

Interest in international survey methodology has recently gained additional traction. The SDGs have a tiering system for indicators which reflects the number and quality of measures available at the global level. Tier 1 indicators, for instance, are conceptually clear, have an established methodology and standards available to use and for which countries produce data on a regular basis. Tier 3 indicators, on the other hand, have no established methodology or standards and they are only now being developed [2]. Against this background, there is an explicit and urgent need to match new household survey tools to the measurement of emerging global topics. Further, the United Nations Statistical Division established an Intersecretariat Working Group on Household Surveys (ISWGHS) in 2015 whose role is to coordinate and harmonize data collection in household surveys. An important part of the work of the ISWGHS is to foster methodological development. Interest in methodological development from household surveys has also been historically studied by specific sectors. For example, the UN Interagency Group for Child Mortality Estimation (IGME) routinely evaluates the performance of different methods of collecting mortality data before using them in estimation procedures [3].

The work of the Improving Coverage Measurement Core Group is particularly relevant in the Maternal, Newborn and Child Health (MNCH) sector, which relies heavily on data on population coverage from household surveys. The papers in this supplement are timely because they reflect on some key indicators and concepts that are becoming increasingly important for the international community and for measurement in household surveys. They typically focus on validating coverage indicators for priority interventions, although some studies start with a very long list of interventions of varying priority. In this paper, we discuss the mechanisms used to develop questions for the DHS and MICS surveys and examine the evidence and circumstances that the programmes use to make decisions about changing global standards. We also provide some key examples of how MNCH-related household survey questions have been adapted over time to take into account new evidence. Finally, we discuss the potential implications of the validation studies in this supplement for household surveys going forward.

## DEVELOPING NEW QUESTIONS FOR DHS AND MICS SURVEYS

DHS and MICS work in rounds, allowing the programmes to periodically update tools to reflect global demands and emerging needs. Questionnaire revision, which is a part of this process, entails considerable effort to develop and test questions that accurately measure the intent of indicators (validity) and for which measures are replicable across settings and over time (reliability).

Deciding what to measure is the first step in question development. Key concepts are clarified and conceptual boundaries are defined. Through this process, concepts are mapped onto suitable indicators or measures which accurately represent the underlying concepts. DHS and MICS have a strong preference for indicators that are applicable across a range of settings and that are meaningful to both national and global audiences. Ultimately, indicators are translated into questions using a multi-stage process.

There are two broad approaches to translating indicators into questions. One is to review validated questions, test them in the context of household surveys and adapt them to the survey instruments. The MICS child discipline module is a product of this approach. Literature reviews showed good validation and extensive use of the Parent-Child Conflict Tactics Scale in the US [4] and across country settings [5], as well as applicability to household surveys, as opposed to many other options that were considered for measuring child discipline. This scale was adopted as a conceptual basis for the MICS module and then was adapted and tested within MICS. This was done chiefly through a global MICS pilot survey where the module was implemented with other MICS modules. Household survey specialists observed the module in the field and extensively debriefed field teams to investigate whether there were specific challenges to implementing the module (including respondents not understanding the questions as intended, asking for questions to be repeated or better explained, having trouble formulating their responses, and feeling uncomfortable when certain questions were asked). Despite the widespread validation and use of the scale in different countries and settings, it was considered crucial to pilot the module in the context of a MICS survey to see how the different topics work in a single instrument and to follow up by monitoring its performance in subsequent surveys. Many sectors also approach the survey programmes with questions that have undergone various levels of validation work but which would need further testing to ensure that they perform well within the context of a MICS or DHS survey. For example, the child functioning module used in MICS6 has undergone extensive validation through cognitive testing and field testing [6,7], both separately and as part of MICS surveys.

When no suitable questions exist to measure an indicator, a second option is to develop them from scratch. The general process is to examine the literature on the topic, review suggestions received from outside experts, and, based on the relevant indicators, define a set of questions that can be adapted for use in MICS or DHS surveys. This process relies heavily on the extensive questionnaire design expertise and field experience within the survey programmes and on close and sustained collaboration between the DHS and MICS programmes in the development of harmonized questionnaires for topics that are of interest to both survey programmes. Following question design, it is the usual practice for MICS and DHS to field test new questions and modules in one or more settings using a number of questionnaire evaluation techniques before these are included country surveys. The field testing includes fieldworker debriefing, behavioural coding of interviews [8], formal validation, and comparison with other studies. In MICS, for example, the early childhood development questions were compared with the Strengths and Difficulties Questionnaire (SDQ) [9] and the Early Development Instrument [10], as summarized in Loizillion et al. [11]. In recent years, cognitive interviewing techniques [12] have been adopted by MICS (and to a lesser extent by The DHS Program) primarily as a means of better understanding question comprehension, although other parts of the response process have also been examined. Cognitive interviewing was used extensively in the development of the Washington Group-UNICEF module on child functioning, which was later incorporated in MICS surveys [6].

It is widely recognized that survey results are affected not only by question wording but also by the placement of questions in a questionnaire. As such, after new questions or modules are designed and validated, they are integrated into existing questionnaires that are tested with other modules under standard field conditions, and the data and experiences from these tests are studied for evidence of their performance (which is discussed in the next section). MICS implements a global pilot survey using all of the new and updated questionnaires, protocols, and tools before the beginning of a new round of surveys to see how all previously validated questions work together in a single instrument. The most recent global MICS pilot took place in Costa Rica, where a new questionnaire for children age 5-17 years was tested, along with a range of new modules and questions (eg, child functioning, water quality testing, social transfers, and assessments of foundational learning skills). At the beginning of the first six rounds of DHS, a large-scale pilot survey of the questionnaires, manuals, and field procedures was conducted. In many cases alternative approaches were incorporated in the pilots. A full-scale pilot survey was not conducted at the beginning of DHS-7 since the questionnaire content was similar to that in DHS-6. During the life course of each round or phase of surveys, additional insights on question performance are gleaned and questions which perform poorly may have to be changed.

The DHS Program developed five new optional questionnaire modules in 2015 (newborn care, including a chlorhexidine submodule; accidents and injuries; male child circumcision; disability; and non-communicable diseases). These modules, along with a substantially revised module on adult mortality, were extensively pilot tested in Ghana [13]. The questionnaire was tested in four languages in 1177 households in urban and rural areas. Interviewers were thoroughly debriefed at the end of the fieldwork. Cognitive interviews were also conducted to identify problems respondents had in understanding the module questions, in being able to answer the questions, and in formulating answers to the questions. Based on the results of the pilot, more than 20 questions in the modules were revised, and the use of the modules in future DHS surveys will be carefully monitored to see if any new problems emerge.

## EVIDENCE AND CIRCUMSTANCES FOR CHANGING GLOBAL STANDARDS IN HOUSEHOLD SURVEYS

Evidence on question performance generally comes from the development stage of the questions (previously outlined) or studies during/after wide-scale implementation. Given that each piece of evidence carries varying levels of rigour and significance, DHS and MICS must consider an entire body of evidence to make global changes to questionnaires, rather than a single source in isolation.

One important source of evidence is experience from the field. DHS and MICS survey specialists provide technical assistance to surveys and observe how questions work across countries. Countries implementing MICS surveys also produce structured pre-test reports wherein they outline implementation issues, including those on question administration. Pre-test reports are also produced for DHS surveys, although some of the reports are abbreviated summaries of key points. These observations are a starting point to accumulate global experience on question quality.

Examining patterns in the data across a large number of countries is another means to interpret question performance. These can include standard data quality checks (which countries produce as part of their survey final reports, for example, in birth histories in DHS and MICS), as well as reports on data quality which the DHS produces during each cycle of surveys [14-16]. These studies are important in that they can reveal patterns that cannot be seen in small-scale testing used to develop questions. In addition, DHS and MICS surveys produce a set of ‘field check’ tables frequently throughout the fieldwork to check on unusual patterns in the data as they are being collected. For example, DHS produces 41 standard field check tables which allow the survey manager to provide immediately feedback to the teams in the field when problems are detected. As DHS and MICS data are cross-sectional, another way of checking on question quality is to determine whether associations conform to theoretical expectations. This is important as correlative evidence is key in the public health literature. These analyses are published in scientific articles and for many indicators show that the data meet overall expectations. Apart from associations, trends are an important part of data quality checking. UNICEF maintains global databases on numerous indicators and populates these using country data, of which DHS and MICS form a large part. Strikingly, numerous indicators show stability and consistency over time in terms of both levels and disaggregation which is a major reason that these were used for MDG monitoring.

DHS and MICS particularly take into account the findings from special validation studies that examine their approaches in general and the accuracy of key indicator measures. These studies should be from a variety of different settings, such as low- and middle-income countries across different regions, preferably using representative samples and resembling DHS and MICS field conditions. With reference to the Improving Coverage Measurement Core Group’s work, studies are well designed and use conditions similar to MICS or DHS. However, certain elements require further consideration. Question design should reflect norms in the survey methodology field. Two studies validated uterotonic use immediately after birth [17,18] and came up with mixed conclusions on the level of sensitivity and specificity. This finding is not surprising, given that the process of delivery is complex, and during delivery, women may undergo processes about which they are unfamiliar or left largely in the dark. Such vague events are not typical candidates for surveys, from a questionnaire design point of view. Additionally, the validation studies here use a ‘gold standard’ for comparison. However, in some instances, these have limited external validity such as the use of health facilities in urban areas or they are not true representations of the ideal phenomenon under question. Statistically, the Improving Coverage Measurement Core Group has also set limits for predictive values and cut-offs that indicate acceptability to include in surveys (eg, an AUC of 0.7). These criteria are, however, somewhat arbitrary (as there is no statistical consensus on the issue). As a result, moving the cut-offs can increase or decrease the number of valid indicators which can lead to different conclusions on what can or cannot be reasonably measured in a household survey. Validation studies thus far have been based on the premise that question performance is largely due to questionnaire design, as well as women’s ability to know or recall events of interest. However, poor predictions and statistical results can also be due to poor training and fieldwork, which need to be considered in the findings.

Country ownership is a key tenet of international development programmes. Household survey programmes must therefore take into account the needs and requirements of national governments, key stakeholders, and funding agencies. While evidence from the above sources provides good inputs for countries to use in designing their questionnaires and tools and while the two survey programmes are able to influence certain outcomes, country-level decision makers ultimately decide the questionnaire content based on a variety of factors in addition to scientific evidence. Countries often want to repeat questions from previous surveys to measure changes over time even if there is evidence that the questions do not perform well. Despite this, DHS and MICS work with countries to ensure that these issues are minimized and that even with departures from the standard questionnaires, the standard indicators can still be calculated.

Apart from country ownership, at the global level, subtle changes in indicators or their metadata can also influence the need to change global standards. These changes may impact indicator numerators and denominators and the algorithms used to calculate the indicators. For example, DHS and MICS have measured sanitation indicators for many years. However, a few years ago, the WHO/UNICEF Joint Monitoring Programme for Water Supply and Sanitation recommended that household surveys also measure whether or not sanitation facilities are shared with other households and use that additional information in deciding on what sanitation facilities are considered to be improved. To this end, an additional question on shared services was included in both DHS and MICS surveys.

## EXAMPLES OF IMPLICATIONS OF VALIDATION STUDIES FOR DHS AND MICS SURVEYS

### Childhood illnesses

Model questionnaires for DHS and MICS surveys have always evolved over time to reflect the most recent scientific evidence, as well as emerging needs and changing priorities. One good example of the historical changes that have taken place is the questions on the prevalence and treatment of symptoms of acute respiratory infection (ARI). DHS and MICS questions on child health originally focused on the three major causes of death for children under five years of age (pneumonia, malaria, and diarrhoeal disease). Since household surveys are not an ideal vehicle for actually diagnosing these diseases in young children, the survey questions asked about symptoms related to these diseases (cough accompanied by rapid breathing, fever, and diarrhoea) during the two-week period preceding the survey.

The earliest DHS surveys (in the 1980s) asked about recent episodes of diarrhoea, treatment-seeking behaviour, and the administration of oral rehydration salts or a home-based sugar, salt, and water solution. In the early 1990s, questions on fever and cough were added to the model woman’s questionnaire. For all children born in the last five years, the mother was asked whether the child had been ill with a cough in the last two weeks and in the last 24 hours, how many days the cough lasted, and whether the child was breathing faster than usual with short, rapid breaths during the illness. Even at that time, it was recognized that those symptoms were not specific to pneumonia, and that they could reflect a common cold, bronchitis, allergies, smoke exposure, or other conditions. The main purpose of those questions was to see if advice or treatment was sought for the child. Additional questions were asked about the type of treatment, but since it was not known what disease the child had, the questions could not determine whether or not the treatment was appropriate. By the time of the fifth round of the DHS surveys (2003-08), it was becoming increasingly clear that additional information on the symptoms of children’s respiratory illness would be needed. At that time, two important changes were made to the questionnaire. First, the question on cough with short, rapid breaths was expanded to include difficulty breathing. Second, a follow-up question was added for children with a cough with rapid breathing to determine whether the mother thought that the rapid or difficult breathing was due to a problem in the chest or a blocked or runny nose. These two additions to DHS were present from MICS3 (roughly 2005-2009). Since that time, only a cough with rapid or difficult breathing that was at least in part chest-related was considered to indicate symptoms of acute respiratory infection. The more recent strong evidence from carefully conducted validation studies in Pakistan, Bangladesh, and Nigeria [19-20], showing that most children identified through survey questions as having the symptoms of ARI do not have pneumonia, has spurred a new round of thinking about the future of these questions in household surveys. However, the fact that these studies showed that the addition of questions on additional symptoms and the use of a video on children with pneumonia moved the needle only marginally closer to determining which children actually have pneumonia means that there is not likely to be any simple fix to the ARI questions.

Although the specific survey questions that are asked are of paramount importance, it is also important to pay attention to the way in which the results are tabulated and interpreted. Even from the beginning, the ARI questions were largely meant to determine whether parents were dealing appropriately with the symptoms rather than whether or not antibiotics were being appropriately administered to sick children. DHS and MICS surveys report on various types of treatment for symptoms of ARI in the standard tables. The inclusion of antibiotics as one type of treatment is not meant to suggest that children with symptoms of ARI should be treated with antibiotics, and the table is not currently interpreted in that way, although some outside agencies or researchers have chosen to interpret the results in different ways. Nevertheless, particularly given increasing resistance to antibiotics, survey reports should take great care to ensure that the reader does not interpret the findings in that way. Out of an abundance of caution, consideration should be given to the possibility of removing antibiotic treatment from the table to avoid confusion. This would be consistent with the conclusions of the three studies in Pakistan, Bangladesh, and Nigeria mentioned above that it is important to continue asking survey questions about the symptoms of ARI and care-seeking behaviour, but that since most young children with those symptoms do not have pneumonia, appropriate treatment cannot be determined.

Attention also needs to be paid to appropriate terminology. DHS has always used the term “symptoms of acute respiratory infection” to refer to the symptoms asked about, even as the questions have changed over time. MICS referred to this indicator as “suspected pneumonia” beginning in 2009, but harmonized terminology with DHS around 2012.

A similar path was taken in the malaria field. In the absence of a blood test for malaria parasites, WHO used to promote presumptive treatment of symptoms of malaria in young children in malaria endemic areas with an appropriate antimalarial medication. However, as the prevalence of malaria has dropped and the availability of rapid diagnostic tests for malaria has risen, WHO changed to its current recommendation to indicate that ‘all cases of suspected malaria should be confirmed using parasite-based diagnostic testing (either microscopy or a rapid diagnostic test) before administering treatment [21]. Treatment solely on the basis of symptoms should only be considered when a parasitological diagnosis is not possible’. When this recommendation was adopted, the standard international indicator on the prompt and effective treatment of fever in children with an antimalarial in malaria endemic countries was discontinued. Consequently, DHS stopped reporting on that indicator, as well. Around the same time, DHS and MICS started asking a question on whether children under five years of age with a recent episode of fever had blood taken from a finger stick for testing. On the advice of the malaria community, the wording of the question was carefully crafted not to specifically mention that the blood was taken for a malaria test, since the mother would often not know the purpose of the test. For a similar reason, a decision was made that DHS, MICS, and Malaria Indicator Surveys would not ask the mother or the caregiver whether a child with fever who had blood taken from a finger stick had a positive or negative result. This decision was informed by a validation study in Zambia that showed poor recall of the result of diagnosis of testing blood from a finger stick [22], as well as a consensus that the provider may prescribe malaria medicine without telling the respondent the test result. If an antimalarial drug is prescribed, the respondent may assume that the test result was positive for malaria even if it wasn’t. In addition, respondents often ask the health provider for antimalarial medicine when their child is ill with fever, and the doctor may prescribe an antimalarial to satisfy the respondent even when antimalarial treatment is not indicated.

### Reference period for childhood illnesses

In addition to the examples given above, some studies have tried to determine whether alternative question wording would improve the measurement of maternal, newborn, and child health questions or perhaps reduce confidence intervals for a given sample size. Some of these studies have examined the effect of changing the length of reference periods for the questions on childhood illnesses and other questions [23-24]. These studies are of vital importance since the confluence of falling fertility rates and the lower prevalence of childhood illnesses in many countries means that there are many fewer cases of childhood illness for a given sample of households. However, the results of these studies to date have been mixed, not providing a solid evidence base for extending the reference period from two weeks to four weeks or more. Moreover, studies that suggest that it is feasible to extend the reference period for one illness (such as ARI) are not necessarily relevant to other illnesses. Since it would be confusing to respondents to have different reference periods for different diseases, it would be desirable to have evidence for all three childhood illnesses that the reference period could be extended without adversely affecting the accuracy of the prevalence and treatment estimates before making a change. If a longer reference period is found to provide accurate results, it would mean that the same degree of precision could be achieved with a smaller sample of households.

### Skin-to-skin contact for newborns

The importance of skin-to-skin contact (SSC) of the newborn with the mother immediately after birth has been well established. Such contact promotes greater respiratory, temperature, and glucose stability, and less stress for the newborn. Based on the growing literature on the utility of SSC, DHS added a question on that topic in recent years. The question was worded ‘Immediately after birth, was (NAME) put directly on the bare skin of your chest?’ However, it was subsequently shown that using just one standard question on SSC does not provide accurate information about what actually occurred around the time of birth [17,25,26]. Based on these ICM Core Group supported studies, the standard DHS question was expanded to two questions:

1. Immediately after birth, was (NAME) put on your chest?
2. IF YES: Was (NAME)’s bare skin touching your bare skin?

MICS began including questions on SSC in round six of the surveys (beginning in 2016), which corresponded to the needs of the Newborn Indicators Technical Working Group. In 2015, MICS reviewed the available questions on SSC and tested an amended set in a field test in Belize. The test also included a photograph of the recommended SSC position, to assist respondents in understanding the SSC concept. Results of cognitive interviews of women in the study indicated that they generally recalled events around the time of delivery, including drying of the newborn and SSC. However, while SSC appeared common, women also said that newborns were often clothed or wrapped, and that SSC lasted only for short periods of time. With these observations in mind, MICS kept the additional question on whether the newborn was wrapped, with the intention of removing these cases from the numerator of the SSC indicator.

The MICS6 questions are as follows:

1. Immediately after birth, was (*name*) put directly on the bare skin of your chest?
  If necessary, show the picture of skin-to-skin position
2. IF YES: Before being placed on the bare skin of your chest, was the baby wrapped up?

While the questions used by both survey programmes are worded slightly different, they are functionally equivalent.

## DISCUSSION

Validation studies are of critical importance when deciding whether or not household survey questions are providing accurate information on what they are seeking to measure. However, validation studies alone may not always provide sufficient evidence on which to base global changes in survey content or question wording. Moreover, although most recent validation studies have been very well designed and carefully implemented, we have several recommendations for how they could be improved to be most useful for household survey programmes.

Household survey programmes base decisions about the validity of questions they use on a variety of evidence, partly because formal validation studies of specific questions are not common. Because questionnaire design is equal parts art and science, DHS and MICS questions are devised by questionnaire experts who have a wealth of experience about what types of questions work well in the context of large-scale, nationally representative household surveys in low- and middle-income countries. First and foremost, the questions are designed so that their intent can be understood by respondents of all types (urban/rural, low education and high education, and respondents of different ages). The questions should be sensitive to cultural norms, to the extent possible. They should be translatable into numerous languages without losing any of their original meaning. Questions asked on the standard questionnaires have generally been pilot tested overall and then pre-tested in each country in all of the languages that are used for interviewing in that country and in both urban and rural areas. During interviewer training, which includes mock interviews and field testing, questions that are difficult to understand in any language are identified. At that point, changes may be made in question wording to improve comprehension, with care being taken not to change the original meaning of the questions.

Feedback received during the course of the fieldwork from interviewers and those monitoring the fieldwork, as well as a full debriefing of interviewers at the end of some surveys, adds to the body of evidence about respondents’ understanding of survey questions. Moreover, even if questions are well understood, they may not produce accurate information either because the respondent does not know the answer, for example, in the case of what happens around the time of birth for institutional births [27], or because of social desirability bias. All of this information is important to help guide future question changes and to inform the interpretation of the survey results.

Cognitive interviewing is increasingly being used by household survey programmes to provide an in-depth look at how people understand survey questions. It is likely that cognitive interviewing will be expanded in the future when designing new questions and reconsidering the wording of some problematic existing questions. Despite the usefulness of cognitive interviewing to gain insights into the understanding of key survey questions, the limitations of cognitive interviewing (for example, lack of generalizability, small samples, and relevance restricted to a particular language or languages) also need to be considered in determining whether or not questions are valid or could be improved.

Formal validation studies supplement the array of methods outlined above, and some examples have been given about how changes in questions or the interpretation of findings have already been made based on the results of validation studies. Even if a particular question is not well validated in one study, it is of concern. However, one study in one location, particularly if it is based on purposive sampling with a small sample of respondents, may not be enough to trigger global changes in question wording or the possibility of deleting some questions. It is necessary to consider the balance of evidence for the validity of an indicator or particular questions from several studies, if available, and the strength of that evidence. It is also important to note that validation studies of survey questions usually compare responses to survey questions to a ’gold standard,’ but the gold standard may itself be imperfect.

The particular recommendations from validation studies should be carefully considered. Should a survey question be retained, changed, or dropped? Can a question that is not well validated be kept if one or more follow-up questions are added to clarify the original response? Are there any other viable sources that could produce reliable data on the same topic or that could provide an accurate estimate of an indicator? If not, are some data (with warts) better than no data at all, as long as the nature of the problems with the questions is well understood and the results are cautiously interpreted? Is a question needed to measure a specific Sustainable Development Goal indicator or another standard indicator (for example, a UNICEF MICS indicator, a UNAIDS indicator, an Infant and Young Child Feeding Practices indicator, or a President’s Malaria Initiative indicator) when there are no other viable sources for such data? A final consideration relates to country ownership of household surveys, as indicated above. We often find that countries want to retain (older) questions in order to measure trends even if it is found that a question is not well validated or the international indicator that is being measured has changed. In such cases, a thoughtful negotiation process may have to take place to ensure that some trend measurement is still possible while changes to ensure accurate measurement in the new survey are accommodated.

Finally, we have a few suggestions to help guide the design of future independent validation studies of survey questions to make their findings most useful to household survey programmes. First, it is important that the basic question wording and translation in a validation study be exactly the same as in DHS or MICS surveys if the study largely aims to validate questions used in those surveys. This is already being done in almost all validation studies. However, it is equally important to try to anchor the study in the context of DHS and MICS surveys in terms of characteristics of respondents, interviewer training, use of the DHS and MICS interviewers’ manuals, field practice, fieldwork procedures, field monitoring, and data quality checks. Otherwise, questions that may be valid in a short survey that focuses on one topic may not be equally valid when integrated into a full DHS/MICS survey. It would be ideal if the questions could be tested in the context of a full DHS or MICS survey, although this may not be practical in most circumstances. It would also be desirable for validation studies to test the inclusion of additional questions, for example, questions on additional symptoms or the use of pill boards or videos, as was done in the ARI validation studies mentioned above. Without this additional information, the results of validation studies leave the survey programmes with the options of retaining or dropping questions, without providing guidance on how the questions might be improved, unless alternative question wording has been included. It would also be desirable to involve DHS and MICS in the development of validation study protocols or alternative strategies for data collection, since some of the alternative methods that might be tested are non-starters for large-scale surveys.

The DHS Program has recently developed a new questionnaire module on maternal health, and the results of all relevant validation studies published to date have been taken into account in decisions about questions to include in this module. A pilot test of the new DHS maternal health module, including cognitive interviewing, is currently underway. DHS and MICS welcome further validation studies of survey questions to provide important information about changes in questions that may be needed or questions that should be considered for deletion because they do not perform well. When questions are well validated in these studies, not only can they be retained, but users of the survey data can have greater confidence in the reliability of questions and the results.
